# Automatic detection of venous air embolism using transesophageal echocardiography in patients undergoing neurological surgery in the semi-sitting position: a pilot study

**DOI:** 10.1007/s10877-020-00568-x

**Published:** 2020-08-18

**Authors:** Tobias R. Rau, Konstanze Plaschke, Markus A. Weigand, Christoph Maier, Christoph Schramm

**Affiliations:** 1 Department of Anesthesiology, Medical Faculty, University of Heidelberg, Im Neuenheimer Feld 110, Heidelberg, 69120 Germany; 2 Department of Medical Informatics, Faculty of Informatics, Heilbronn University of Applied Sciences, Max-Planck-Str. 39, Heilbronn, 74081 Germany; 3 Institute of Medical Biometry and Informatics, University of Heidelberg, Im Neuenheimer Feld 130.3, Heidelberg, 69120 Germany

**Keywords:** Neuroanaesthesia, Sitting position, Venous air embolism, Transesophageal echocardiography, Automatic detection of venous air embolism

## Abstract

Neurological surgery in the semi-sitting position is linked with a pronounced incidence of venous air embolism (VAE) which can be fatal and therefore requires continuous monitoring. Transesophageal echocardiography (TEE) provides a high sensitivity for the intraoperative detection of VAE; however, continuous monitoring with TEE requires constant vigilance by the anaesthesiologist, which cannot be ensured during the entire surgical procedure. We implemented a fully automatic VAE detection system for TEE based on a statistical model of the TEE images. In the sequence of images, the cyclic heart activity is regarded as a quasi-periodic process, and air bubbles are detected as statistical outliers. The VAE detection system was evaluated by means of receiver operating characteristic (ROC) curves using a data set consisting of 155.14 h of intraoperatively recorded TEE video and a manual classification of periods with visible VAE. Our automatic detection system accomplished an area under the curve (AUC) of 0.945 if all frames with visible VAE were considered as detection target, and an AUC of 0.990 if frames with the least severe optical grade of VAE were excluded from the analysis. Offline-review of the recorded TEE videos showed that short embolic events (≤ 2 min) may be overseen when monitoring TEE video manually. Automatic detection of VAE is feasible and could provide significant support to anaesthesiologists in clinical practice. Our proposed algorithm might possibly even offer a higher sensitivity compared to manual detection. The specificity, however, requires improvement to be acceptable for practical application. Trial Registration: German Clinical Trials Register (DRKS00011607).

## Introduction

If the blood pressure of veins in a surgical site is less than the atmospheric pressure, air can enter the venous system and cause a lung air embolism (VAE) [1]. Air emboli increase the pressure in the pulmonary circulation due to an obstruction of pulmonary arterioles and a constriction of the intrapulmonary vasculature [1, 2]. Depending on the total amount of air and the speed of infusion, VAE can be fatal [3] with an estimated lethal volume of 3–5 ml/kg [4].

There exist mainly two methods for the intraoperative detection of VAE in the clinical practice [4]: (i) the precordial doppler ultrasound and (ii) the transesophageal echocardiography (TEE). Of these two the TEE has higher sensitivity and can be seen as diagnostic standard for the detection of VAE [4, 5].

A major disadvantage of the non-automatic detection, however, is the requirement of uninterrupted visual monitoring of the TEE loops during the surgical procedure, which cannot be provided to 100% by the anesthesiologist, who has a variety of other tasks during surgery. Further, an existing approach to the automated detection of VAE in the TEE depends on the definition of a region of interest by the anesthesiologist [6]. The interaction required with such an automatic detection system will likely decrease its acceptance.

**Automatic** monitoring of TEE images without interaction of the anesthesiologist, however, could make a substantial contribution to the detection of VAE, e.g. during neurosurgical procedures in the semi-sitting position, a surgical procedure which is linked with a pronounced incidence of VAE [7].

We therefore analyzed the ability to detect VAE in the intraoperative TEE by a fully automatic detection system which is based on a statistical model and does not require interaction with the anesthesiologist.

## Materials and methods

### Study design and patient enrollment

After approval by the institutional ethics committee (approval number: S-611/2016), data collection of this prospective study was conducted from February 2, 2017 until June 6, 2017 in the Department of Anesthesiology of the Heidelberg University Hospital (Germany). The trial was registered at the German Clinical Trials Register (DRKS00011607).

Patients were included if they were at least 18 years old, underwent a neurosurgical procedure at the posterior cranial fossa, the cerebellopontine angle or the upper cervical spine and had no contraindications for TEE. All patients signed informed consent. A total of 39 patients were included in the study in order to record a sufficiently large dataset.

All patients underwent surgery in the semi-sitting position. The pre- and intraoperative anesthesia management was performed according to a standard protocol of the clinical department. After induction of general anesthesia with sufentanil, propofol, and rocuronium, anesthesia was maintained with continuous application of propofol, desflurane or sevoflurane. Two large bore peripheral venous catheters, an arterial catheter, a central venous line, and a Foley catheter were inserted. High quantities of crystalloid solutions were administered starting at the induction of anesthesia to reach a state of slight hypervolemia in each patient. Before placing the patient in the sitting position, a complete TEE-examination was performed to exclude persistent foramen ovale or other right-to-left shunts. During surgery, patients were continuously monitored for the occurrence of VAE with the mid-esophageal right-ventricular in- and outflow-tract view of the TEE.

The TEE examinations were performed with the approved CX50 ultrasound system and the 2D TEE-transducer × 7-2t (Philips, Hamburg, Germany).

Upon recognition of intraoperative VAE in the TEE by the study personnel, a bilateral compression of the jugular veins was applied and the neurosurgeons immediately coagulated and waxed the sites of air entry. If necessary, further measures were taken, including the anti-Trendelenburg movement of the operating table, the application of a crystalloid bolus and the increase of positive end-expiratory pressure (PEEP).

### Data collection

For all included patients, age, gender, weight, height, ASA classification, indication for surgery, and surgery time was noted.

The loops of the intraoperative TEE monitoring were saved as video files to an external hard drive using an Epiphan DVI2USB 3.0™ frame grabber. The recorded TEE videos had a resolution of 628 × 458 pixels and were compressed with either Lagarith Lossless codec (5 patients) or the more efficient Motion JPEG Video codec (34 patients). In total, 155.14 h of TEE videos were recorded. The average frame rate was 38 frames per second.

If air bubbles where observable in TEE during surgery, the exact time of these periods was noted and the amount of visible air was qualitatively estimated and classified into three degrees of severity: grade 1 (minor amount of air), grade 2 (medium amount of air), and grade 3 (major amount of air). For air bubbles to be rated as embolic, a continuous flow of visible air was required, i.e. singular, isolated bubbles were not considered in our detection. In addition to the visual grading, clinical significance of all VAE events, which were detected during surgery, was classified according to the Tübingen VAE grading scale [8] (see Table 1).

**Table 1:** Tübingen scale of VAE detection [8]

Grade	Definition
0	No air bubbles visible on TEE
I	Air bubbles visible on TEE
II	Air bubbles visible on TEE with decrease of end-tidal carbon dioxide ≤ 3 mmHg
III	Air bubbles visible on TEE with decrease of end-tidal carbon dioxide > 3 mmHg
IV	Air bubbles visible on TEE with decrease of end-tidal carbon dioxide > 3 mmHg and decrease of mean arterial pressure ≥ 20% or increase of heart rate ≥ 40% (or both)
V	Same as grade IV causing hemodynamic instability requiring cardiopulmonary resuscitation

Periods affected by video superimpositions (e.g. due to an alarm message), repositioning of the TEE ultrasound probe, or intermittent freezing of the TEE video transfer were identified and excluded from further analysis.

### Algorithm for the automated detection of VAE

The detection algorithm for VAE was implemented in Python v3.6 using OpenCV [9], NumPy [10] and SciPy [11].

#### Detection of the cardiac phase

Continuous calculation of the mean grayscale value within a rectangular region of 367 × 377 pixels in the TEE video provides a cyclic signal, which reflects cardiac periodicity. We filtered this signal using a high-pass Butterworth filter of second order at the critical frequency of 5 Hz resulting in a sequence $\left\{{x}_{n}\right\}$ of smoothed average pixel intensities. For every timepoint n an embedded vector was formed from the sequence $\left\{{x}_{n}\right\}$ as follows, based on the theorem of Takens [12]:

$${\overrightarrow{x}}_{n}\text{ } = \text{ }\left(\begin{array}{ccccc}{x}_{n-12}& {x}_{n-9}& {x}_{n-6}& {x}_{n-3}& {x}_{n}\end{array}\right)$$

In an initialization step, a reference cycle $\left\{{\overrightarrow{x}}_{n}\right\}$ of the embedding vector trajectory covering one cardiac period was extracted and subdivided into 15 cardiac phase segments. During subsequent analysis, an instantaneous cardiac phase value was assigned to each frame. It was defined as the phase segment number associated with the corresponding reference cycle embedding vector. The latter was identified as the reference vector with minimum Euclidean distance to the current embedding vector.

#### Detection of statistical outliers

In analogy to [13], the images of each cardiac phase were modeled with a gaussian model, which is defined by the mean and the standard deviation of the grayscale value for every pixel. Therefore, for every pixel position and separately for every phase, a rolling mean µ and standard deviation σ of the last 30 images of the corresponding cardiac phase were calculated. In order to suppress detections in dark areas of the image with very low variation, the minimum value for the standard deviation was set to the 65% quantile of all values for σ in the TEE image.

A pixel was classified as being part of an air embolus if its grayscale value I exceeded the mean by at least k standard deviations:

$$I-\mu >k\sigma$$

The optimal free parameter k was estimated in a preliminary experiment. For every frame in the TEE videos the total number of pixels classified as air embolus was recorded.

### Statistics

The TEE videos were analyzed according to 2.3 and the number of pixels classified as air by the algorithm was recorded for every frame defining a time series. At times with a repositioning of the TEE ultrasound probe or after freezing of the TEE video transfer, a reinitialization of the detection algorithm (i.e. reference cycle and pixel statistics) was triggered.

The recorded time series of number of detected air pixels was filtered using a rolling median with a window size of 140 frames in order to be robust against short outliers. In order to model the background noise floor, the 15% quantile of the number of detected air pixels for the first 140 frames after every reinitialization of the detection algorithm was calculated and subtracted from the rolling median. The resulting time-series was compared to the time-matched sequence of the visual VAE grading reference by means of receiver operating characteristic (ROC) curves. Periods with a disturbance in the TEE video or a repositioning of the TEE ultrasound probe were excluded from this analysis. Likewise, consecutive images, with normalized cross correlation of 0.999 or higher (indicating temporal freezing in the video transfer) were not considered.

## Results

### Patient characteristics and recorded videos

Demographic and surgical characteristics of the 39 included patients of this study are shown in Table 2.

**Table 2:** Surgical and demographic characteristics

	Mean (min – max)
Age [years]	50.7 (19 – 81)
Gender	m = 19, f = 20
Weight [kg]	84.6 (43 – 170)
Height [cm]	171.8 (153 – 195)
ASA 1/2/3/4	1/22/16/0
Surgery duration [h]	4.2 (2,1 – 7.6)

*m* male, *f* female, *ASA* classification of physical status according to the american society of anesthesiologists

In total 155.14 h of TEE videos were recorded; 6.29 h were marked as air emboli with optical grade 1; 1.58 h were marked as air emboli with optical grade 2, and 0.10 h were marked as air emboli with optical grade 3.

### Incidence of VAE and sensitivity of *manual* VAE detection

During the study, 180 intervals of intraoperative VAE were detected. Seventy-five events occurred directly after intravenous administration and were self-limiting within a short period of time (typically < 1.5 min). These events were not considered for the calculation of the incidence of VAE. The remaining 105 VAE events occurred in 28 of the 39 included patients (72%). The Tübingen VAE grading scale for these events is summarized in Table 3.

**Table 3:** Air embolic events detected by Tübingen VAE grading scale

Tübingen VAE grading scale	Number of events
Sum of all gradings	105
I	31
II	13
III	2
IV	2
V	0
No grading available*	57

*If VAE was detected during a second offline review process, the Tübingen VAE grading scale could not be determined. Clinical grading was also not available for one additional event, which was created by splitting an intraoperatively detected event

A second iteration of review of the TEE videos focused on inspection of false alarms triggered by the automatic VAE detection algorithm. This targeted scan identified 56 additional VAE events originally missed during surgery. Fifty-four of these events where of short length (≤ 2 min). The other two events lasted 2.6 min and 6.2 min respectively. Both contained phases with little air alternating with phases showing sporadic air bubbles without continuous flow in the TEE. Data of end-tidal carbon dioxide (EtCO2) for VAE events detected by the post-surgical screening was not available.

The targeted post-surgical screening of the recorded TEE videos revealed that of 180 actual VAE events only 124 had been detected during surgery. The sensitivity of VAE detection by manual monitoring of the TEE can therefore be estimated to be 69%.

Of the 124 VAE events, which were detected during surgery, 17 events had a Tübingen VAE grading scale of II or greater, indicating a decrease of EtCO2. This means that VAE detection by monitoring of the EtCO2 would achieve a sensitivity of 9%.

### Preliminary experiment for *automatic* VAE detection

**Fig. 1:**
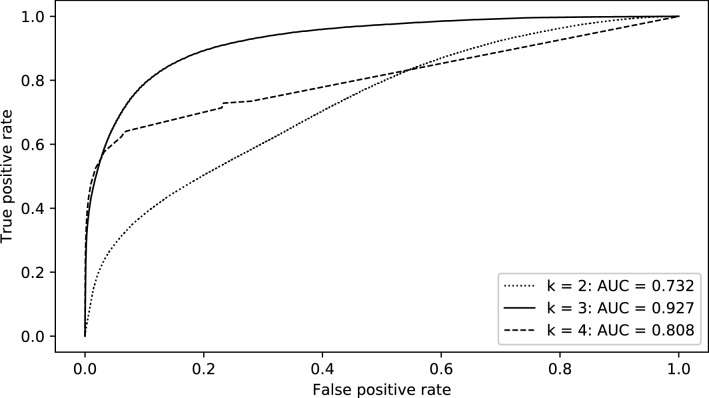
ROC curves for the detection of frames with observable VAE for different values of k

In order to estimate the optimal parameter k of the automatic detection algorithm, a ROC analysis was performed for different values of k using only the videos of the 12 patients with the longest periods of VAE during surgery (Fig. 1). This analysis yielded the highest AUC (0.927) for k = 3 compared to k = 2 (AUC = 0.732) and k = 4 (AUC = 0.808). Therefore, the parameter k was set to 3 for further analysis over the full dataset of TEE videos.

### Evaluation of *automatic* VAE detection

**Fig. 2:**
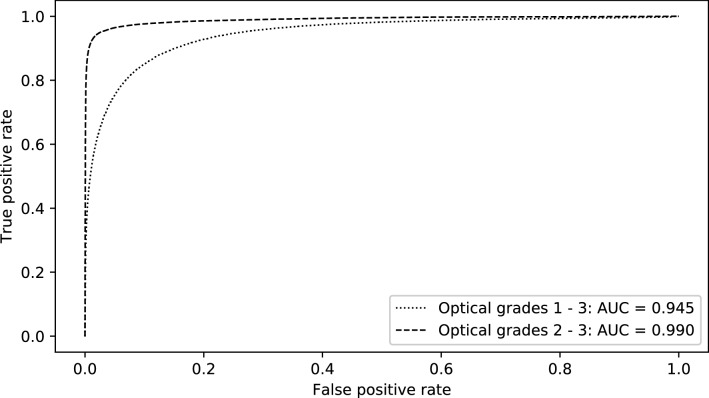
ROC curves for the detection of frames with observable VAE. For the analysis of frames with at least optical grade 2, all frames with optical grade 1 were excluded. Requiring a detection of 90% of phases with visible air within 3 s where false positive frames with a distance of maximum 38 frames (i.e. 1 s) are considered as a single alarm event, the detection algorithm would lead to a false alarm rate of 1.5 alarms per minute. Requiring a detection of 90% of the phases with at least optical grade 2 the algorithm results in a false alarm rate of 0.1 alarms per minute

If all frames with a continuous flow of air bubbles were to be considered as detection target, the ROC analysis of the automatic detection algorithm would lead to an AUC of 0.945, as shown in Fig. 2. An exclusion of frames with optical grade 1 from the analysis yields an AUC of 0.990 (Fig. 2). Frames during phases with sporadic air bubbles but without continuous flow of emboli were always considered as non-targets, i.e. as frames without visible air bubbles.

In a practical monitoring setting, the described system would operate at a false alarm rate of 1.5/min if detection of 90% of all VAE events was required within a response time of 3 s (i.e. sensitivity forced to 0.9). In this calculation, multiple detections less than 1 s apart (corresponding to 38 frames) are counted as one single alarm. The number of false positive alarms reduces to 0.1/min if only VAE events of medium and severe grade are considered as targets.

## Discussion

The reported incidence of air embolism during neurological surgery in the semi-sitting position in the literature range from 2–76% [14, 15]. This wide range is partly explained by varying definitions of VAE [16]. Compared with studies, which also define VAE by the appearance of air bubbles in the TEE, the incidence of 72% observed in this study is in the upper range of reported incidences of 26–76% [5, 8, 15–17]. However, the incidence of VAE reported in this study might also be overestimated as in contrast to these studies we also included embolic events which were detected during targeted offline review after surgery.

The targeted offline review of the recorded TEE videos identified 56 additional VAE events. A majority of these events might actually be caused by intravenous administration of solutions due to their short and self-limiting duration. These events can therefore be assumed to be of low clinical significance. Unfortunately, data of EtCO2 to confirm this assumption retrospectively was not available.

17 of 124 VAE events (9%), which were detected by TEE during surgery, where associated with a decrease of EtCO2. This is in accordance with the lower sensitivity of EtCO2 compared to TEE [18]. It should be noted, that a drop of EtCO2 may even be prevented due to immediate actions taken in response to the detection of VAE by TEE monitoring. This might decrease the sensitivity of VAE detection by monitoring of EtCO2 in our study.

Our algorithm for the automatic detection of VAE in TEE was able to detect VAE with an AUC of 0.945 for all frames with visible air bubbles, and an AUC of 0.990 for all frames with a medium or high amount of visible air bubbles. Since especially short embolic events can easily be overlooked by the anesthesiologist when monitoring the TEE during surgery, we conclude that the automatic detection algorithm could provide significant support in clinical practice. In addition, the automatic detection does not require any interaction with the anesthesiologist and is expected to be robust to the usage of alternative standard views in TEE as it only depends on statistical assumptions about the cyclic nature of TEE images.

The optical grading used in this study was dependent on the subjective classification of the amount of visible air bubbles in the TEE video. We did not use a quantitative cutoff to differentiate between the three predefined classes. Consequently, we can’t exclude a certain degree of overlap and incidental inconsistent assignment of frames between adjacent categories. In order to improve consistency, we limited ourselves to distinguishing only coarsely between three optical grades of severity. Moreover, all videos were classified by the same person. Despite the given limitations we are confident that this classification represents a useful measure of visible air und is adequate as a reference for performance assessment of the suggested detection method.

Certainly, the current false alarm rate of 1.5/min at a sensitivity of 90% for events of any grade of severity would not be acceptable in a clinical application. Still, please note that this enforced sensitivity of 90% is set about 20% higher than the estimated sensitivity of manual VAE detection. Furthermore, the significantly lower false alarm rate of 0.1/min for events of medium and major severity (again at 90% sensitivity) indicates that the algorithm is focusing on the right target. Still, the specificity of the proposed algorithm is currently too low for application in clinical routine. False alarms were typically triggered by the false positive detection of ventricular or valvular structures as air bubbles. Reasons for such false positive detections included accidental changes of the TEE probe position or dysrhythmia. Consequently, our group is currently pursuing several alternative approaches and refinements which mainly aim at improving detection specificity. If we succeed in overcoming this limitation, it seems achievable that fully automatic intraoperative monitoring of the TEE video stream can improve VAE detection sensitivity.

## Criticism and outlook

There are different possible improvements to our algorithm. First of all, it should be noted that a gaussian model is a very rough estimation of the speckle pattern in ultrasound images, which is typically modeled with a Rayleigh distribution [19]. Therefore, considering the Rayleigh distribution could potentially improve the model. Furthermore, usage of a mixture of gaussians may lead to a better detection of VAE.

The process of detecting the cardiac phase of every frame of the TEE videos could possibly be improved by considering also morphological features of the images. Even though the current algorithm works well in identifying images of the same cardiac phase, they typically don’t match exactly, but are rather shifted in different directions. This fact might at least partially be explained by ventilation of the patient. A compensation of these shifts might significantly increase the ability of our algorithm to detect VAE.

Another promising approach is the application of more recent machine learning algorithms such as deep neural networks. Such algorithms have already successfully been applied to various fields of medicine, such as for example ophthalmology [20] or dermatology [21].
